# Effectiveness of a WeChat Mini Program–Based Intervention on Promoting Multiple Health Behavior Changes Among Chinese Patients With Cardiovascular Diseases in Home-Based Rehabilitation: Randomized Controlled Trial

**DOI:** 10.2196/66249

**Published:** 2025-06-03

**Authors:** Yanping Duan, Wei Liang, Lan Guo, Huimin Zhan, Chunli Xia, Huan Ma, Borui Shang, Yanping Wang, Min Yang, Shishi Cheng

**Affiliations:** 1 Department of Sports and Health Sciences, Hong Kong Baptist University, Hong Kong, China (Hong Kong); 2 School of Physical Education, Shenzhen University, SYB 326, 3688 Nanhai Road, Nanshan District, Shenzhen, 518060, China, 86 15217940540; 3 Cardiac Rehabilitation Center, Guangdong Cardiovascular Institute, Guangdong Provincial People’s Hospital (Guangdong Academy of Medical Sciences), Guangzhou, China; 4 Cardiac Intensive Care Department, Cardiovascular Disease Institute, Guangdong Provincial People's Hospital (Guangdong Academy of Medical Sciences), Southern Medical University, Guangzhou, China; 5 Department of Social Sciences, Hebei Sport University, Shijiazhuang, China; 6 Department of Sports Science and Physical Education, The Chinese University of Hong Kong, Hong Kong, China (Hong Kong)

**Keywords:** digital health, physical activity, nutrition, cardiac rehabilitation, Health Action Process Approach, HAPA, WeChat, intervention, health behavior change, health behavior, China, cardiovascular, cardiovascular disease, CVD, home-based rehabilitation, domiciliary rehabilitation, randomized controlled trial, behavioral theories, healthy lifestyle, multiple health behavior change

## Abstract

**Background:**

WeChat mini program–based interventions grounded in behavior change theories show promise in promoting and maintaining healthy lifestyles among patients with cardiovascular diseases (CVDs) after hospital discharge. However, limited randomized controlled trials have evaluated the effectiveness of such interventions among Chinese patients with CVDs in a home-based rehabilitation context.

**Objective:**

This study aimed to assess the effectiveness of a 10-week WeChat mini program–based intervention on multiple health behaviors, including moderate to vigorous physical activity (MVPA), fruit and vegetable consumption (FVC), integrated lifestyle indicator (ie, meeting both MVPA and FVC recommendations), psychosocial resources (intrinsic and extrinsic) of behavior change, and health-related outcomes (ie, depressive symptoms and perceived quality of life) among Chinese patients with CVDs.

**Methods:**

This study recruited 166 outpatients from a cardiac rehabilitation center in China. After screening for eligibility and randomization, 124 participants (mean age 41.60, SD 13.48 years; 61.3% female) were allocated to either (1) the intervention group, which received a 10-week health program based on the Health Action Process Approach, or (2) a waitlist control group, which received no intervention and maintained their usual lifestyle. Both groups completed assessments at baseline, postintervention (10 weeks), and 3 months postintervention. Data were analyzed using generalized linear mixed models in IBM SPSS 28.0.

**Results:**

Significant time-by-group interaction effects were observed for MVPA (*F*_2, 122_=6.68; *P*=.002), FVC (*F*_2, 122_=18.68; *P*<.001), integrated lifestyle indicator (*F*_2, 122_=13.83; *P*<.001), intrinsic (*F*_2, 122_=11.49; *P*<.001) and extrinsic psychosocial resources (*F*_2,1 22_=5.35; *P*=.006) for MVPA, intrinsic resources for FVC (*F*_2, 122_=12.66; *P*<.001), and perceived quality of life (*F*_2, 122_=6.99; *P*=.001). The intervention group showed significant improvements in these outcomes compared to the control group, with medium-to-large effect sizes for behavior-related outcomes (*d*=0.57‐0.88), and small-to-medium effect sizes for psychosocial and health-related outcomes (*d*=0.28‐0.52). However, no significant effects were found for extrinsic resource for FVC (*F*_2, 122_=1.37; *P*=.26) or depressive symptoms (*F*_2, 122_=0.44; *P*=.64). Sensitivity analyses confirmed the robustness of the primary findings.

**Conclusions:**

The 10-week Healthy Action Process Approach–based WeChat mini program intervention effectively improved MVPA, FVC, integrated lifestyle indicator, psychosocial resources of behavior change, and health-related outcomes among Chinese patients with CVDs. This intervention provides a valuable addition to rehabilitation strategies aimed at promoting long-term health and activity in cardiac patients following hospital discharge.

## Introduction

### Background

Cardiovascular diseases (CVDs), the leading cause of mortality globally, encompass a variety of disorders that impair the heart and blood vessels (eg, coronary heart disease, cerebrovascular disease, and peripheral arterial disease) [1,2]. In China, approximately 330 million individuals are afflicted with CVDs, constituting 46.9% of total annual deaths [3]. Cardiac rehabilitation, a critical component of comprehensive CVD management, has been demonstrated to be effective in preventing disease progression and recurrence [4]. It provides patients with CVD with guidance on adopting a healthy lifestyle, emphasizing the importance of engaging in adequate physical activity (PA; eg, ≥150 min of moderate-to-vigorous PA [MVPA] per week) and maintaining a nutritious diet (eg, ≥5 servings of fruit and vegetables per day) [5]. The success of cardiac rehabilitation significantly depends on its incorporation into the patients’ routine postdischarge [6,7]. Nonetheless, extensive research has demonstrated the challenges patients encounter in applying these lifestyle recommendations and educational outcomes into their lives after hospitalization [8,9]. Therefore, patients require both intrinsic and extrinsic resources, which can be effectively provided through extended rehabilitation aftercare programs during their recovery at home [10,11].

Recent advancements have positioned digital health as a forefront medium in delivering health services through the internet and associated technologies, including computers, smartphones, and wearable devices [12]. Research has demonstrated the efficacy of digital health interventions in promoting PA and fruit and vegetable consumption (FVC), thereby supporting the transition to healthier lifestyles for patients with CVDs following their discharge [7,12-15]. In China, the majority of home-based digital health cardiac rehabilitation programs have focused on knowledge dissemination, education, and telephone consultations [11,16,17]. However, few have implemented an integrated, individualized approach that includes educational, cognitive, and psychological components. Moreover, these interventions often address only a single behavior, such as PA or diet, without considering the potential synergistic effects of promoting multiple behaviors simultaneously.

This study used the Health Action Process Approach (HAPA) framework to guide the interventions for MVPA and FVC [18]. The HAPA framework suggests that behavior change is a dynamic, ongoing process, encompassing 2 distinct phases. The motivational phase is crucial for enhancing risk perception (eg, the likelihood of experiencing a CVD), action self-efficacy (eg, confidence in one’s ability to engage in FVC effectively), and outcome expectancies (eg, anticipated benefits and consequences from participating or not participating in adequate MVPA) [18,19]. These factors contribute to strengthening intentions to change behaviors, such as committing to perform ≥150 minutes of MVPA per week or consuming ≥5 portions of fruit and vegetables daily [18,19]. Upon forming such intentions, individuals progress to the volitional phase, where they can use various self-regulatory strategies to initiate and sustain behavior. These strategies include action planning (eg, determining the specifics of MVPA engagement), coping planning (eg, strategies to sustain MVPA amid challenges), maintenance self-efficacy (eg, confidence in continuing to consume sufficient fruit and vegetables despite obstacles), recovery self-efficacy (eg, confidence in resuming MVPA after lapses), and action control (eg, ongoing self-monitoring to prevent relapse) [18-21]. Additionally, fostering perceptions of social support plays a crucial role in maintaining behaviors and preventing relapse [11,18,20]. Overall, cardiac rehabilitation patients require both intrinsic (eg, intention, self-efficacy, planning, action control) and extrinsic resources (eg, behavior-specific social support) to adopt and sustain a healthy lifestyle following hospital discharge [11,18].

In our previous pilot study, the efficacy of a HAPA-based, internet-delivered intervention targeting PA and FVC among patients with coronary heart disease undergoing home-based rehabilitation was investigated [11]. The findings indicated that the intervention surpassed the control condition in enhancing PA, FVC, psychological resources of behavioral change (eg, self-efficacy and planning for PA and FVC, and FVC-related social support), and health-related outcomes (eg, quality of life). The intervention demonstrated significant efficacy, with η^2^ effect sizes ranging from 0.06 to 0.43. Moreover, a notable increase in the adoption of an integrated healthy lifestyle (ie, adhering to both MVPA and FVC recommendations) was observed, with 40% of participants in the intervention group versus 10% in the control group meeting both behavioral recommendations. Nevertheless, the study was limited by its design, only implementing pretest and posttest measurements with a lack of follow-up observation for the residual effects of the intervention. Additionally, the intervention content did not incorporate action control, a critical component for sustaining behavioral changes. The intervention also had a short duration of only 4 weeks for each health behavior (PA/FVC) and exclusively included patients who could access the internet via a computer.

WeChat, a widely popular social media platform in China with over 800 million active users [22], was selected as the intervention platform due to its broad accessibility, seamless integration into daily life, and user-friendly design. Unlike conventional approaches (eg, smartphone apps), WeChat mini programs do not require installation or uninstallation, which significantly enhances convenience and promotes sustained user engagement [23]. These unique features make WeChat an ideal platform for delivering health interventions, ensuring that patients can easily incorporate the program into their routines without additional barriers. Although WeChat mini programs have shown promise for various digital health interventions, their use in cardiac rehabilitation for patients with CVD remains limited.

### Purpose and Hypotheses

The primary purpose of this study was to evaluate the effectiveness of a 10-week intervention delivered through a WeChat mini program, targeting MVPA and FVC among Chinese patients with CVD undergoing home-based rehabilitation. Four hypotheses were proposed: (1) the intervention group (IG) would demonstrate greater improvements in MVPA and FVC behaviors compared to the waitlist control group (CG); (2) the IG would be more likely to meet the World Health Organization (WHO) recommended guidelines for both MVPA and FVC (ie, the integrated health lifestyle), in comparison with the CG; (3) the IG would achieve greater progress in the psychosocial determinants of behavioral change (ie, intrinsic and extrinsic resources), relative to the CG; and (4) the IG would experience improvements in health-related outcomes (ie, depressive symptoms and perceived quality of life), compared to the CG.

## Methods

### Ethical Considerations

The study adhered to the Declaration of Helsinki and was approved by the Research Ethics Committee of Hong Kong Baptist University (FRG2/17-18/099). Participants signed an informed consent form. As an incentive, participants received a 50 RMB (US $6.94) telephone recharge card for participating in and completing the 3-wave data collection. Data were anonymized and deidentified.

### Study Design, Participants, and Procedure

This randomized controlled trial (RCT) included 2 groups: an IG and a CG. Both groups received usual care, but the IG additionally participated in a 10-week WeChat mini program–based health intervention, while the CG was asked to maintain their normal lifestyle during the study period. The health intervention was designed based on the HAPA framework, focused on promoting MVPA and FVC over the 10-week period. Following the completion of data collection, the CG was granted access to the same intervention materials. Data were collected at 3 time points: baseline (T1), immediately postintervention (T2), and at a 3-month follow-up (T3).

The study targeted outpatients diagnosed with CVDs, including coronary artery disease, hypertension, and heart failure. However, patients with congenital heart disease were excluded due to the unique nature of their condition, which requires different management strategies not addressed by our intervention. Inclusion criteria mandated that participants must: (1) be aged 18 to 75 years; (2) possess unrestricted physical mobility and cardiac function at entry; (3) not have other diseases, including diabetes, or allergies/intolerances to fruits and vegetables (as these factors may confound the intervention effects); (4) be free from cognitive or mental disorders; (5) have successfully completed the Physical Activity Readiness Questionnaire screening or received physician approval for participation; (6) not be engaged in other concurrent programs promoting a healthy lifestyle (eg, physical exercise and/or nutrition); (7) possess adequate Chinese reading and writing skills; and (8) have access to WeChat and the internet through a smartphone.

The sample size was calculated using G*Power 3.1 software, drawing on data from our previous pilot study [11]. To ensure a robust analysis of the within-between interaction, 122 participants (61 per group) were deemed necessary. This estimation accounted for a medium effect size (Cohen *f*) of 0.33 on PA and FVC, a significance level of .05, a statistical power of 80%, and an attrition rate of 20% [11]. Participants with CVDs were recruited randomly using a random number table by the research team, consisting of a physician and 2 nurses from the Cardiology Department of Guangdong Provincial People’s Hospital in Guangzhou, China. Of the 186 individuals approached during initial recruitment, 166 expressed interest and completed the qualification examination. Moreover, 124 eligible participants were invited to sign an informed consent form and register online through our WeChat mini program. Upon registration, participants were randomized into either the intervention or control group in a 1:1 ratio. This randomization process was managed by an independent research assistant using a simple list on the mini program’s back end, ensuring no involvement in the intervention, data collection, or outcome evaluation. Due to ethical guidelines, participant blinding was not feasible; however, the intervention was delivered automatically via the WeChat mini program (meaning that the research implementers were not aware of the participants’ group assignments), and outcome assessors were blinded to the group assignment, allowing for a double-blinded design [24]. Additionally, participants were grouped into separate WeChat groups based on their intervention allocation to facilitate engagement through weekly reminders. The study was conducted between August 2020 and April 2023.

### Intervention Content

The multiple health behavior change intervention, spanning 10 weeks, was administered through a WeChat mini program titled “eHealth Homeland” (健康家园网站 in Chinese). This program was structured into 2 distinct modules. The first module featured a comprehensive 10-week intervention designed to address social-cognitive factors related to MVPA and FVC, grounded in the HAPA framework. Participants were encouraged to engage with this module weekly for a duration of 20 minutes. The second module served as a centralized data repository, facilitating the collection of prior health behaviors and the management of incentive activities. It granted participants unfettered access to their records throughout the intervention period.

In Module 1, the intervention targeted specific components based on the HAPA for MVPA and FVC over the 10-week period as follows: in week 1, the focus was on risk perception and outcome expectancies; in week 2, action self-efficacy and goal setting; weeks 3 and 4 concentrated on enhancing action self-efficacy and introducing maintenance self-efficacy with action planning; week 5 extended this by incorporating coping planning; week 6 continued with maintenance self-efficacy and coping planning; weeks 7 and 8 added recovery self-efficacy and social support; and weeks 9 and 10 culminated in reinforcing maintenance self-efficacy, recovery self-efficacy, coping planning, social support, and action control. To promote the initiation and maintenance of behavior change, the intervention used a series of behavior change techniques (BCTs) [25], including education on the risks and consequences of unhealthy lifestyles (BCTs 5.1‐5.6), verbal persuasion to boost confidence in performing and sustaining healthy behaviors (BCTs 15.1‐15.2), and encouragement of self-monitoring of health behaviors and their outcomes (eg, emotional experiences, weight status, blood pressure, and perceived physical health; BCTs 2.1‐2.7).

Module 2 was structured into 4 main sections: Home Page, Check-In, Archive, and Open Forum. Within the Home Page section, participants had the opportunity to review the content of each session completed in Module 1 [26]. The Archive section allowed for the examination of aggregated data from Module 1, divided into 4 subsections: “My Behavior Record,” which provided graphs offering individualized feedback on PA and FVC for each week; “My Action Plans,” detailing the action plans devised for PA and FVC; “My Coping Plans,” outlining the coping strategies for PA and FVC; and “My Diary,” which included reflective diaries on PA and FVC. Further details regarding the intervention content are available in our previously published protocol paper [26].

### Measures

#### Primary Outcomes: Health Behaviors and Integrated Lifestyle Indicator

MVPA assessment used the abbreviated Chinese version of the International Physical Activity Questionnaire (intraclass correlation coefficient=0.77) [27]. Participants were required to detail both the frequency (occurrences per week) and duration (minutes per occurrence) of engaged vigorous (eg, brisk bicycling, intense swimming) and moderate (eg, carrying light loads, cycling at a normal pace) physical activities over the last week. The overall MVPA metric for each participant, quantified in minutes weekly, was derived by summing all pertinent responses [27,28].

FVC over the preceding week was evaluated using 4 specific items: “raw vegetables,” “fruits,” “raw fruit and vegetable juice,” and “cooked or steamed vegetables” (Cronbach α=0.71) [29]. Participants were instructed to record their average daily intake for each category, quantifying the number of servings or glasses of fruit and vegetables consumed (with options ranging from 0, 0.5, 1, 1.5, …, to 5 or more). This process was facilitated by pictorial instructions. The aggregate daily FVC was determined by totaling the servings from all pertinent categories [26,29].

The integrated lifestyle indicator evaluates the confluence of 2 health behaviors by measuring adherence to recommendations for PA and FVC. As per the WHO directives, participants are advised to engage in a minimum of 150 minutes of moderate-intensity PA weekly, or 75 minutes of vigorous-intensity PA, or an equivalent combination thereof. Additionally, the consumption of 5 servings of fruit and vegetables daily is recommended [30,31]. In this study, participants’ adherence to health behavior recommendations was classified into 3 categories: 0=did not meet any behavioral recommendations, 1=met one behavioral recommendation, and 2=met both behavioral recommendations [11].

#### Secondary Outcomes: Intrinsic and Extrinsic Resources and Health-Related Outcomes

The intrinsic resources related to MVPA and FVC included intention, self-efficacy, planning, and action control. These components were measured through a package of items derived from previous studies [11,32-36]. Specifically, intention for each behavior was evaluated using 3 items (eg, “I intend to undertake vigorous physical activity for at least 30 min daily over five days a week, or a minimum of 150 minutes weekly,” “I intend to consume no less than five servings of fruits and vegetables daily”; Cronbach α=0.63‐0.67) [11,21,26,32]. Self-efficacy for each behavior was measured using 5 items (eg, “I am confident in my ability to engage in MVPA for at least 30 min, five days a week,” “I am confident in my ability to consume five servings of fruits and vegetables daily, despite possible obstacles”; Cronbach α=0.93‐0.96) [11,21,33]. Planning was evaluated through 6 items, divided into 3 for action planning (eg, “I have meticulously planned my physical activities for the next month,” “I have meticulously planned my fruit and vegetable intake for the next month”) and 3 for coping planning (eg, I have devised strategies for overcoming difficult situations to adhere to my intentions for MVPA,” I have planned for adequate fruit and vegetable consumption, even in unforeseen circumstances”; Cronbach α=0.93‐0.94) [11,33,34]. Action control was evaluated through 6 items, asking participants about their awareness, effort, and self-monitoring in maintaining MVPA and FVC (eg, “I regularly check whether I am engaging in sufficient MVPA,” “I regularly ensure that I am consuming enough fruits and vegetables”; Cronbach α=0.89‐0.91) [26,35,36]. Responses were collected using visual analogue scales rather than Likert scales due to their intuitive design, precision in capturing responses, and reduced participant dropout rate [37]. The overall score of intrinsic resources for behavior change was calculated as the mean of the scores across each component, with a scoring range of 1 to 5. Higher scores indicate more substantial intrinsic resources for behavior change [11].

The extrinsic resource, specifically social support, associated with MVPA and FVC, was assessed using 3 items for each behavior (Cronbach α=0.86‐0.87) [11,26,38]. Items included statements such as “My partner/family/friends and acquaintances encourage me to engage in adequate MVPA” or “My partner/family/friends and acquaintances encourage me to intake sufficient fruit and vegetables.” Responses were recorded on a visual analogue scale with a range from 1 to 4, where higher scores reflect greater external support for behavior change.

For health-related outcomes, depressive symptoms were assessed using the Chinese version of the Center for Epidemiological Studies-Depression (CES-D) scale [39]. Participants responded to a prompt, “In the past week, how often have I felt…” followed by 10 items, such as “...I was bothered by things that usually don’t bother me.” Responses were captured on a 4-point Likert scale, ranging from 0 (“rarely or none of the time, <1 day”) to 3 (“most or all of the time, 5‐7 days”; Cronbach α=0.75). The total score, ranging from 0 to 30, was calculated by summing the responses, with higher scores indicating more severe depressive symptoms [39].

For perceived quality of life, the Chinese short form of the WHO Quality of Life-BREF (WHOQOL-BREF) was used [19,40]. This instrument comprises 9 items: 2 assess general quality of life (eg, “How would you rate your quality of life?”) and 7 pertain to the physical health subdomain (eg, “To what extent does physical pain prevent you from doing what you need to do?”; Cronbach α=0.70). The overall perceived quality of life score was derived from the average of these 9 items, ranging from 1 to 5, where higher scores denote better perceived quality of life [40].

### Additional Measures: Demographic Information

In addition to the primary and secondary outcomes collected via the WeChat mini program through the 3-wave data collection process, demographic information (including age, gender, fertility status, educational background, living conditions, occupational status, and measurements of body weight and height for calculating BMI) was collected at the registration stage.

### Statistical Analyses

Data analysis was performed using SPSS software (version 28.0; IBM Corp). Independent *t* tests and *Χ*^*2*^ tests were used to assess the effectiveness of the randomization process. When significant group differences at baseline were noted, such variables were adjusted for as covariates in subsequent analyses [41]. The investigation of intervention effects followed the intention-to-treat principle, complemented by per-protocol analysis for sensitivity assessment [42]. To address missing data, the multiple imputation method using chained equations was used, with the exception of dropouts, for which the baseline-observation-carried-forward strategy was applied [19,43].

The assessment of intervention effects on various outcome measures over time was performed through generalized linear mixed models, incorporating time, group, and their interaction as fixed effects, and individuals as random effects. Model selection favored an unstructured covariance structure based on the −2log likelihood, Akaike, and Bayesian information criteria, using a restricted maximum likelihood method. For post hoc analyses, the least significant difference method was preferred to mitigate the risk of type II errors and preserve study power, particularly in studies with fewer than 3 comparison groups, over other adjustment methods (eg, Bonferroni) [44,45]. *Χ*^*2*^ tests were also used for post hoc analyses. To further enhance our understanding and inform the development of future interventions, a dropout analysis was conducted. This analysis assessed the differences in baseline characteristics between participants who dropped out and those who completed the program, at postintervention and follow-up stages. Independent samples *t* tests and chi-square tests were used to examine these differences. The statistical significance threshold for all analyses was established at *P*<.05 (2-tailed).

## Results

### Randomization Check and Sample Characteristics

An examination of the randomization procedure revealed no significant variances in baseline descriptive characteristics, including age, gender, fertility status, educational background, living situation, occupational status, BMI, and BMI category, alongside all outcome indicators (*P*=.16‐0.93), across both the intervention and the waitlist control groups. These findings substantiate the efficacy of the randomization process.

**Figure 1.**
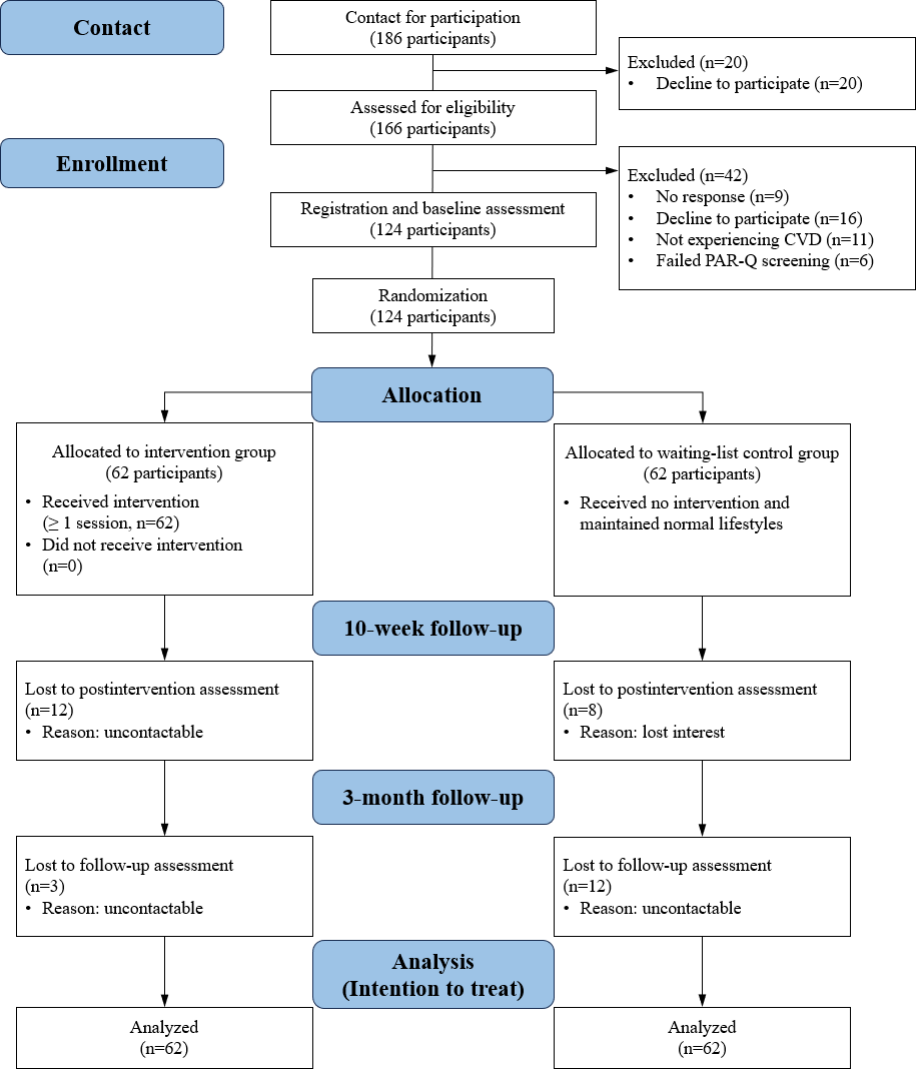
The CONSORT diagram of study process. CONSORT: Consolidated Standards of Reporting Trials; CVD: cardiovascular disease; PAR-Q: Physical Activity Readiness Questionnaire.

Table 1 summarizes the descriptive characteristics of the study sample. The study included 124 eligible participants who completed the baseline assessment, comprising 76 females (61.3%) and 48 males (38.7%) with an average age of 41.6 (SD 13.48) years. Most participants (81/124, 65.3%) reported having children, and 91.1% (113/124) had achieved an educational level of at least secondary school. The prevalent living situation (84/124, 67.7%) was with others, such as family or partners, with 59.7% (74/124) of the sample being employed. Additionally, 31.5% (39/124) of the participants were classified as overweight or obese. Using an intention-to-treat analysis approach, all participants who completed the baseline assessment were included in the final analysis (Figure 1).

**Table 1.** Descriptive characteristics of the study sample at baseline (N=124).

Variable	Total (N=124)	Intervention group (n=62)	Control group (n=62)	*P* value
Age (years), mean (SD)	41.60 (13.48)	41.13 (13.11)	42.06 (13.93)	.70
**Gender, n (%)**				.71
Female	76 (61.3)	37 (59.7)	39 (62.9)	
Male	48 (38.7)	25 (40.3)	23 (37.1)	
**Fertility status, n (%)**				.57
Have child	81 (65.3)	39 (62.9)	42 (67.7)	
No child	43 (34.7)	23 (37.1)	20 (32.3)	
**Educational background, n (%)**				.35
Primary and below	11 (8.9)	5 (8.1)	6 (9.7)	
Secondary school	59 (47.6)	26 (41.9)	33 (53.2)	
College and above	54 (43.5)	31 (50)	23 (37.1)	
**Living situation, n (%)**				.70
Living alone	40 (32.3)	19 (30.6)	21 (33.9)	
Living with others	84 (67.7)	43 (69.4)	41 (66.1)	
**Occupational status, n (%)**				.71
Employed	74 (59.7)	38 (61.3)	36 (58.1)	
Unemployed	50 (40.3)	24 (38.7)	26 (41.9)	
BMI, kg/m^2^, mean (SD)	22.58 (4.62)	22.62 (4.80)	22.46 (4.54)	.78
**BMI category, n (%)**				.55
Underweight	20 (16.1)	8 (12.9)	12 (19.4)	
Healthy weight	65 (52.4)	35 (56.5)	30 (48.4)	
Overweight and obese	39 (31.5)	19 (30.6)	20 (32.3)	
**Primary outcomes**				
MVPA^a^ (min/week), mean (SD)	124.23 (80.31)	120.81 (78.52)	127.65 (82.56)	.64
FVC^b^ (portions/day), mean (SD)	4.01 (1.69)	3.81 (1.45)	4.20 (1.90)	.21
***Integrated lifestyle indicator, n (%)***				.85
Unhealthy lifestyle^c^	65 (52.4)	34 (54.8)	31 (50)	
Unhealthy lifestyle^d^	35 (28.2)	17 (27.4)	18 (29)	
Healthy lifestyle^e^	24 (19.4)	11 (17.7)	13 (21)	
**Secondary outcomes, mean (SD)**				
Internal resources for MVPA	2.82 (0.85)	2.78 (0.85)	2.85 (0.85)	.64
External resource for MVPA	2.34 (1.01)	2.33 (0.98)	2.35 (1.04)	.93
Internal resources for FVC	2.81 (0.85)	2.87 (0.92)	2.75 (0.78)	.45
External resource for FVC	2.63 (1.03)	2.76 (1.05)	2.49 (1.00)	.16
Depressive symptoms	12.27 (5.03)	12.52 (5.05)	12.03 (5.04)	.59
Perceived quality of life	3.17 (0.86)	3.08 (0.88)	3.26 (0.84)	.25

a MVPA: moderate-to-vigorous physical activity.

b FVC: fruit and vegetable consumption.

c Unhealthy lifestyle: meeting no behavior recommendations (performing ≥150 min of MVPA per week and consuming ≥5 portions of fruit and vegetables per day).

d Unhealthy lifestyle: meeting only one behavior recommendation.

e Healthy lifestyle: meeting both behavior recommendations.

### Intervention Effects on Health Behaviors and Integrated Lifestyle Indicator

**Figure 2.**
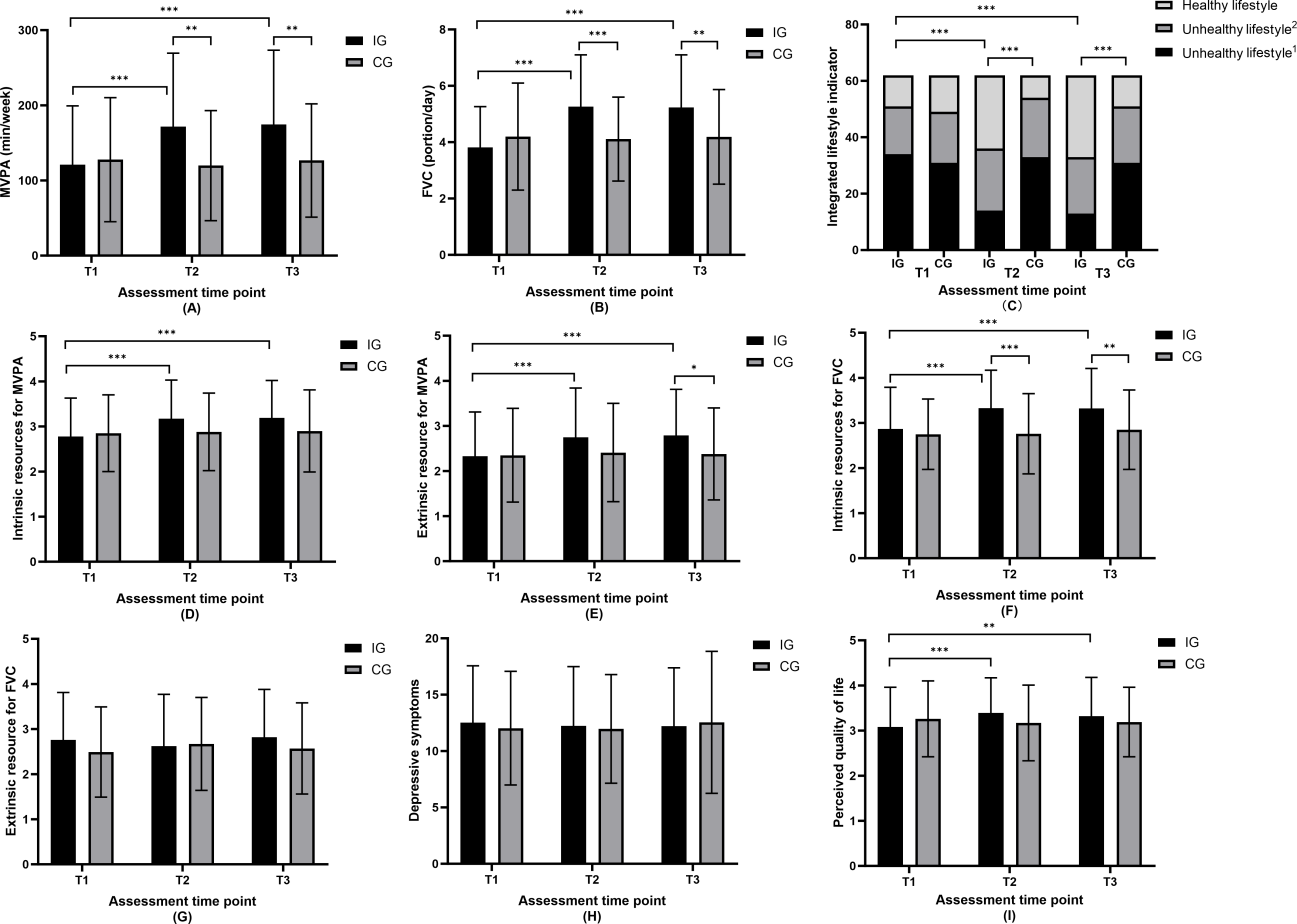
Mean values of intervention and control groups at 3 time points. (A) MVPA; (B) FVC; (C) integrated lifestyle indicator; (D) intrinsic resources for MVPA; (E) extrinsic resource for MVPA; (F) intrinsic resources for FVC; (G) extrinsic resource for FVC; (H) depressive symptoms; (I) perceived quality of life. CG: control group; FVC: fruit and vegetable consumption; IG: intervention group; MVPA: moderate-to-vigorous physical activity; Unhealthy lifestyle 1 refers to meeting no behavior recommendations; Unhealthy lifestyle 2 refers to meeting only one behavior recommendation.

Table 2 presents the effects of the intervention on primary outcomes, including weekly MVPA, daily FVC, and an integrated lifestyle indicator. Figures 2A-2C describe the changes of these primary outcomes from T1 to T3 for 2 groups. The analyses indicated that both MVPA (*P*=.002) and FVC (*P<*.001) improved significantly over time, exhibiting significant interaction effects between the IG and CG. For the integrated lifestyle indicator, the percentage of IG participants adhering to a healthy lifestyle increased markedly from 17.7% at baseline to 41.9% at T2 and 46.8% at T3, whereas in the CG, this proportion declined from 21% at baseline to 12.9% at T2, and eventually to 17.7% at T3. The results of the generalized linear mixed models confirmed a significant interaction between time and group concerning the integrated lifestyle indicator (*P<*.001). Subsequent post hoc tests on time effects revealed medium to large effect sizes for MVPA (Cohen *d*_T1T2_=0.57, Cohen d_T1T3_=0.60), FVC (Cohen *d*_T1T2_=0.88, Cohen d_T1T3_=0.85), and the integrated lifestyle indicator (Cohen *d*_T1T2_=0.68, Cohen d_T1T3_=0.76) in the IG relative to the CG.

**Table 2.** Results of the intervention effects examination on health behaviors and integrated lifestyle indicator (N=124).

Outcome	T2^a^		T3^b^		Time × group^c^		Time^c^		Group^c^	
	Intervention group	Control group	Intervention group	Control group	*F/χ*^2^ test (*df*)	*P* value	*F/χ*^2^ test (*df*)	*P* value	*F/χ*^2^ test (*df*)	*P* value
MVPA^d^, mean (SD)	171.73 (97.67)	119.76 (73.15)	174.56 (98.81)	126.55 (75.39)	6.68 (2, 122)	.002	5.02 (2, 122)	.008	6.06 (1, 122)	.02
FVC^e^, mean (SD)	5.26 (1.84)	4.11 (1.49)	5.23 (1.87)	4.19 (1.68)	18.68 (2, 122)	<.001	16.54 (2, 122)	<.001	4.84 (1, 122)	.03
**Integrated lifestyle indicator, n (%)**										
Unhealthy lifestyle^f^	14 (22.6)	33 (53.2)	13 (20.9)	31 (50.0)	13.83 (2)	<.001	9.66 (2)	.008	18.03 (1)	<.001
Unhealthy lifestyle^g^	22 (35.5)	21 (33.9)	20 (33.3)	20 (33.3)						
Healthy lifestyle^h^	26 (41.9)	8 (12.9)	29 (46.8)	11 (17.7)						

a T2: postintervention assessment.

b T3: 3-month follow-up assessment.

c Type III *F* tests were used for MVPA and FVC, while Wald chi-square tests were used for integrated lifestyle indicator.

d MVPA: moderate-to-vigorous physical activity (min/week).

e FVC: fruit and vegetable consumption (portions/day).

f Unhealthy lifestyle: meeting no behavior recommendations (performing ≥150 min of MVPA per week and consuming ≥5 portions of fruit and vegetables per day).

g Unhealthy lifestyle: meeting only one behavior recommendation.

h Healthy lifestyle: meeting both behavior recommendations.

### Intervention Effects on Intrinsic and Extrinsic Resources and Health-Related Outcomes

Table 3 presents the effects of the intervention on secondary outcomes, including intrinsic and extrinsic resources for MVPA and FVC, depressive symptoms, and perceived quality of life. Figures 2D-2I illustrate the changes of these secondary outcomes from T1 to T3 for the 2 groups. The results revealed significant time and group interaction effects on intrinsic and extrinsic resources related to MVPA and FVC (all *P*<.006), except extrinsic resource for FVC (*P*=.26). For 2 health-related outcomes, only perceived quality of life demonstrated a significant interaction effect (*P*=.001), in contrast to depressive symptoms (*P*=.64). Subsequent post hoc tests revealed small to medium effect sizes for changes in intrinsic and extrinsic resources for MVPA (Cohen *d*_T1T2_=0.46, Cohen *d*_T1T3_=0.49 for intrinsic resources; Cohen *d*_T1T2_=0.41, Cohen *d*_T1T3_=0.46 for extrinsic resource) and FVC (Cohen *d*_T1T2_=0.52, Cohen *d*_T1T3_=0.50 for intrinsic resources; Cohen *d*_T1T2_=−0.13, Cohen *d*_T1T3_=0.06 for extrinsic resource), along with depressive symptoms (Cohen *d*_T1T2_=−0.06, Cohen *d*_T1T3_=−0.06) and perceived quality of life (Cohen *d*_T1T2_=0.37, Cohen *d*_T1T3_=0.28), favoring the IG over the CG.

**Table 3.** Results of the intervention effect examination on intrinsic and extrinsic resources and health-related outcomes (n=124).

Outcome	Mean (SD) at T2^a^		Mean (SD) at T3^b^		Time × group^c^		Time^c^		Group^c^	
Intervention group	Control group	Intervention group	Control group	*F* test (*df*)	*P* value	*F* test (*df*)	*P* value	*F* test (*df*)	*P* value
Intrinsic resources for MVPA^d^	3.17 (0.86)	2.88 (0.86)	3.19 (0.83)	2.90 (0.91)	11.49 (2, 122)	<.001	16.62 (2, 122)	<.001	1.45 (1, 122)	.23
Extrinsic resource for MVPA	2.75 (1.09)	2.41 (1.09)	2.79 (1.02)	2.38 (1.02)	5.35 (2, 122)	.006	7.66 (2, 122)	<.001	2.30 (1, 122)	.13
Intrinsic resources for FVC^e^	3.33 (0.84)	2.76 (0.89)	3.32 (0.89)	2.85 (0.88)	12.66 (2, 122)	<.001	17.29 (2, 122)	<.001	7.31 (1, 122)	.008
Extrinsic resource for FVC	2.62 (1.15)	2.67 (1.03)	2.82 (1.06)	2.57 (1.01)	1.37 (2, 122)	.26	0.53 (2, 122)	.59	0.99 (1, 122)	.32
Depressive symptoms	12.23 (5.26)	11.97 (4.82)	12.21 (5.18)	12.55 (6.30)	0.44 (2, 122)	.64	0.22 (2, 122)	.81	0.03 (1, 122)	.87
Perceived quality of life	3.39 (0.78)	3.17 (0.84)	3.32 (0.86)	3.19 (0.77)	6.99 (2, 122)	.001	2.36 (2, 122)	.10	0.16 (1, 122)	.69

a T2: postintervention assessment.

b T3: 3-month follow-up assessment.

c Type III test.

d MVPA: moderate-to-vigorous physical activity (min/week).

e FVC: fruit and vegetable consumption (portions/day).

### Sensitivity Analysis and Dropout Analysis

The sensitivity analyses conducted with a per-protocol approach revealed significant time and group interaction effects for all variables (all *P*<.03), except the extrinsic resource for FVC (*P*=.66) and depressive symptoms (*P*=.17). This indicates consistency with the results of the primary intention-to-treat analyses (Multimedia Appendix 1).

The participant dropout rate was 16.1% (20/124) from T1 to T2, and 14.4% (15/104) from T2 to T3. Cumulatively, the dropout rate from T1 to T3 was 28.2% (35/124). No significant difference in dropout rates was observed between groups at T2 (*Χ*^2^_^1^_=0.95; *P*=.33). However, at T3, the CG exhibited a descriptively higher dropout rate compared to the IG (6% vs 22.2%). A meaningful statistical analysis could not be performed for T3 due to fewer than 5 dropouts in the IG. Significant differences in baseline information between completers and dropouts at T2 were found in age (*P*<.001), educational background (*P*=.02), MVPA (*P*=.007), and extrinsic resource of MVPA (*P*=.047). At T3, the integrated lifestyle indicator was the only baseline characteristic showing a significant difference between completers and dropouts (*P*=.001). More details are available in Multimedia Appendix 2.

## Discussion

### Principal Findings

This study aimed to investigate the efficacy of a 10-week WeChat mini program intervention in promoting multiple health behaviors among Chinese patients with CVD undergoing home-based rehabilitation. The findings substantially corroborated the proposed research hypotheses.

The anticipated effects of the intervention on MVPA, FVC, and an integrated lifestyle indicator (ie, adhering to both MVPA and FVC recommendations) were confirmed (hypotheses 1 and 2). Participants in the IG exhibited prominent enhancements in both MVPA and FVC over the study period compared to those in the control group (hypothesis 1). These results concerning the modification of multiple health behaviors align with findings from our preliminary pilot study [11], as well as studies involving individuals with type 2 diabetes [46], individuals with metabolic syndromes [47,48], and cancer survivors [49]. Notably, previous digital interventions targeting health behavior improvements exhibit variability in their delivery mode (eg, computer-based, smartphone-based, website-based, mini program–based), reflecting their adaptability to user preferences and technological advancements [7]. The WeChat mini program, a premier social media application in China, stands out for its extensive accessibility, adaptability, and user engagement, making it a potential tool for health promotion efforts [48,50,51]. Despite the efficacy of WeChat mini program–delivered interventions for enhancing PA, dietary adherence, and smoking cessation, investigations into their effectiveness on multiple health behaviors in cohorts with CVD remain sparse [48,50,51].

It is worth noting that previous studies targeting individual health behaviors (eg, PA or diet) often overlooked the interconnected nature of diverse health behaviors [52,53]. Health behaviors are not entirely autonomous; insights into the determinants of one behavior can often be generalized to others that are similar. Research has demonstrated that these analogous behaviors tend to co-occur and may have synergistic or cumulative effects (eg, the observed gateway effect between PA and healthy eating) [53-55]. Addressing multiple health behaviors simultaneously can lead to unforeseen health advantages and outcomes [52,55]. Furthermore, evidence has consistently emphasized the benefits of interventions promoting multiple health behaviors in enhancing health promotion, amplifying health gains, and decreasing health care expenditures compared to interventions that focus on a single health behavior [54-56]. Our study revealed that participants who engaged in a 10-week HAPA-based intervention exhibited a higher rate of adherence to both MVPA and FVC recommendations relative to those in the CG (hypothesis 2). This finding illuminates the potential and applicability of WeChat mini program–based interventions in simultaneously promoting multiple health behaviors in patients with CVD, particularly within the context of home-based rehabilitation, thereby contributing to the expanding evidence base in support of digital health strategies.

The effects of our intervention on intrinsic (comprising intention, self-efficacy, planning, and action control) and extrinsic (social support) resources for behavior change (hypothesis 3) were significant for 3 of the 4 measures. Aligning with existing literature, our intervention enhanced intrinsic resources related to MVPA and FVC, underlining the critical role of personal intrinsic factors in promoting behavior change [11,48,57,58]. These findings also add contemporary empirical support to literature. However, contrary to our initial findings from a previous pilot study [11], a significant increase in social support for FVC was not observed. This discrepancy may be attributed to several factors, with the predominant explanation being the impact of the COVID-19 pandemic. During the new normal of the pandemic, participants in our study might have encountered challenges in securing fruit and vegetables (eg, declined supply, increased price), which may have impeded the intended effects of our intervention on the extrinsic resource related to FVC [59]. In other words, the broader social context during this period likely interfered with the true efficacy of the intervention [60]. In contrast to MVPA, adopting and sustaining FVC behavior requires more substantial efforts, including both individual psychological motivators and financial commitments. This implies that future health behavior interventions should take more social factors into account (eg, the role of community-based food support programs, peer-coaching models, or environmental influences). Overall, the majority of hypothesis 3 was supported.

Regarding the intervention effects on health-related outcomes (hypothesis 4), our study demonstrated that participants in the IG exhibited substantial improvements in their perceived quality of life, which aligns with the findings of our preliminary pilot study [11] and corroborates evidence from prior research involving individuals with chronic conditions [61]. These improvements are anticipated due to the critical role that adequate PA and a healthy diet play in promoting well-being, both of which are pivotal in significantly improving the individuals’ quality of life [62,63]. However, the intervention did not yield significant benefits regarding depressive symptoms; no notable advancements were observed in the IG following the intervention or at the subsequent follow-up. This finding may be attributed to the high prevalence of depressive symptoms among CVD patients at the beginning of the study, with 62.9% (78 of 124 participants) reporting scores of 10 or higher—the threshold for mild depressive symptoms—highlighting the significant mental health concerns within this group [39]. Moreover, the mental health conditions of patients may have deteriorated further during the COVID-19 pandemic [64,65]. Addressing depressive symptoms in CVD patients may require the future development of long-term, comprehensive behavioral interventions that incorporate guidance, information, stress management, and relaxation techniques. This approach is further validated by the success of another intervention targeting rehabilitation patients with myocardial infarction [66]. Overall, hypothesis 3 was partially supported.

Finally, the sensitivity analyses corroborated the primary analyses, supporting the robustness of the findings. In terms of dropout rates, this study exhibited a notably lower dropout rate at postintervention assessment (16.1%) compared to our previous pilot study (27.2%) [11]. Additionally, the cumulative dropout rate from baseline to follow-up assessments was significantly lower than those observed in prior app-based interventions for chronic patients (28.2% vs 40%) [67]. This improvement might be attributed to the advantageous delivery mode and strategies used in this study (eg, check-ins, interactive discussion forums, multiple reminders, and incentives). It is worth noting that older age, lower educational levels, higher levels of baseline MVPA, and sufficient external resources for MVPA were critical characteristics of dropouts at postintervention assessments. Moreover, dropouts during the follow-up period were significantly less likely to adopt unhealthy lifestyles compared to those who completed the program. This suggests that individuals of older age and lower educational backgrounds may face greater challenges in participating in our intervention program. Furthermore, the program might not appeal to individuals who are already sufficiently active and have adequate social support, as they may deem the intervention unnecessary. Conversely, those struggling to adopt a healthier lifestyle may require additional support during the follow-up period. It means that individuals who face barriers to maintaining healthier behaviors, despite their efforts, may need ongoing support to achieve long-term behavior change. Both the HAPA and self-determination theory emphasize that external support, such as encouragement from family and peers, is crucial in overcoming these challenges [18,68]. Future interventions should take these aspects into account.

### Strengths and Limitations

This study offers significant theoretical and practical contributions to the development of future digital health interventions aimed at fostering multiple health behaviors. The incorporation of the HAPA framework and BCTs established a solid theoretical base for assessing intervention efficacy. The comprehensive sensitivity analyses and dropout analyses reinforced the external validity of our findings.

Despite the methodological strengths and the profound implications of the research, there are several limitations that warrant attention. First, the use of self-reported measures may cause recall bias and social desirability bias. Participants may have overreported their PA and FVC due to the desire to appear more health conscious. Although we used validated instruments to reduce these biases, we acknowledge that self-reported data might still have influenced the accuracy of the results. Future studies could benefit from incorporating objective measures, such as accelerometers or food diaries, to complement self-reports and provide a more accurate assessment of behavior. Second, the study sample was relatively younger (mean age 41.6, SD 13.48 years) compared to other CVD populations. This may be due to the exclusion of participants with diabetes mellitus, a common comorbidity in older individuals with CVD, which likely contributed to the younger average age of the sample. Additionally, the higher proportion of women in the sample may reflect gender differences in health care–seeking behavior, as well as a greater willingness among women to participate in health-related interventions. These factors should be considered when interpreting the findings and their generalizability to other populations. The applicability of these results to other CVD populations (eg, older patients or those with different conditions), as well as across diverse cultural contexts, requires further investigation. Moreover, the provision of monetary incentives for participation may raise concerns about bias in evaluating the intervention effects. Furthermore, the waitlist control design, while methodologically sound, may introduce some placebo effects attributable to differential attention between study groups. Future research could use active control conditions (eg, comparable digital interventions) to more precisely account for these potential effects. Additionally, the health-related outcomes in our study, limited to depressive symptoms and perceived quality of life, may not fully capture the comprehensive health benefits for patients with CVD. Future research would benefit from incorporating additional cardiometabolic biomarkers (eg, fasting glucose, glycated hemoglobin, triglycerides, high-sensitivity C-reactive protein, interleukin-6, tumor necrosis factor alpha). Finally, the 3-month follow-up period in this study provides initial evidence of behavioral maintenance, while longer-term assessments (6‐12 mo) would be valuable to evaluate sustained intervention effects.

### Conclusions

In summary, this study demonstrated the effectiveness of interventions delivered via a WeChat mini program, grounded in the HAPA, in facilitating multiple health behavior changes among Chinese patients with CVD during home-based rehabilitation. The majority of the study hypotheses were confirmed, indicating that such digital health interventions are promising in enhancing the scope of extended rehabilitation strategies for patients with CVD, enabling them to maintain an active and healthy lifestyle postdischarge. This study contributes novel insights to the field of health behavior change research and offers both theoretical and practical guidance for the development and implementation of future digital health interventions aimed at health promotion.

## Supplementary material

10.2196/66249 Multimedia Appendix 1 Results of sensitivity analysis.

10.2196/66249 Multimedia Appendix 2 Results of dropout analyses.

10.2196/66249 Checklist 1 CONSORT-eHEALTH (V 1.61).

## Abbreviations

BCT: behavior change technique
CG: control group
CVD: cardiovascular disease
FVC: fruit and vegetable consumption
HAPA: Health Action Process Approach
IG: intervention group
MVPA: moderate to vigorous physical activity
PA: physical activity
RCT: randomized controlled trial
T1: baseline
T2: postintervention
T3: 3-month follow-up
WHO: World Health Organization
