# Supplementary material for: Effectiveness of a WeChat Mini Program–Based Intervention on Promoting Multiple Health Behavior Changes Among Chinese Patients With Cardiovascular Diseases in Home-Based Rehabilitation: Randomized Controlled Trial
Source: J Med Internet Res. 2025 Jun 3;27:e66249. doi: 10.2196/66249 (PMC12151454; doi:10.2196/66249)
Supplement: Multimedia Appendix 1 [file jmir-v27-e66249-s001.docx]

**Table S1.** Results of Sensitivity test using a per-protocol analysis strategy (n = 89).

| **Outcome** | **Time×group^1^** | | **Time^1^** | | **Group^1^** | |
| --- | --- | --- | --- | --- | --- | --- |
|  | ***F/χ*^2^ test (*df*)** | ***P* value** | ***F/χ*^2^ test (*df*)** | ***P* value** | ***F/χ*^2^ test (*df*)** | ***P* value** |
| MVPA | 8.36 (2, 87) | <0.001 | 5.04 (2, 87) | 0.09 | 1.83 (1, 87) | 0.18 |
| FVC | 18.62 (2, 87) | <0.001 | 16.49 (2, 87) | <0.001 | 5.68 (1, 87) | 0.019 |
| Integrated lifestyle indicator | 17.17 (2) | <0.001 | 11.78 (2) | 0.003 | 13.38 (1) | <0.001 |
| Intrinsic resources for MVPA | 9.50 (2, 87) | <0.001 | 20.04 (2, 87) | <0.001 | 2.01 (1, 87) | 0.15 |
| Extrinsic resource for MVPA | 3.73 (2, 87) | 0.028 | 6.12 (2, 87) | 0.03 | 1.36 (1, 87) | 0.25 |
| Intrinsic resources for FVC | 12.32 (2, 87) | <0.001 | 16.95 (2, 87) | <0.001 | 5.97 (1, 87) | 0.017 |
| Extrinsic resource for FVC | 0.42 (2, 87) | 0.66 | 0.55 (2, 87) | 0.58 | 0.42 (1, 87) | 0.52 |
| Depressive symptoms | 1.80 (2, 87) | 0.17 | 0.56 (2, 87) | 0.58 | 0.03 (1, 87) | 0.87 |
| Perceived quality of life | 5.98 (2, 87) | 0.004 | 3.96 (2, 87) | 0.023 | 0.14 (1, 87) | 0.71 |

Note. ^1^Type III tests; MVPA = moderate-to-vigorous physical activity (min/week); FVC = fruit and vegetable consumption (portion/day); SD = standard deviation; df = degree of freedom; IG = intervention group; CG = waiting-list control group; T2 = post-intervention assessment; T3 = follow-up assessment.
