# Supplementary material for: Effectiveness of a WeChat Mini Program–Based Intervention on Promoting Multiple Health Behavior Changes Among Chinese Patients With Cardiovascular Diseases in Home-Based Rehabilitation: Randomized Controlled Trial
Source: J Med Internet Res. 2025 Jun 3;27:e66249. doi: 10.2196/66249 (PMC12151454; doi:10.2196/66249)
Supplement: Multimedia Appendix 2 [file jmir-v27-e66249-s002.docx]

**Table S2.** Differences in the baseline characteristics of the completers and dropouts at different time points.

| **Variable** | **Post-intervention assessment (T2)** | | | **Follow-up assessment (T3)** | | |
| --- | --- | --- | --- | --- | --- | --- |
|  | **Completers**  **(n = 104)** | **Dropouts**  **(n = 20)** | ***P* value** | **Completers**  **(n = 89)** | **Dropouts**  **(n = 15)** | ***P* value** |
| **Demographics** |  |  |  |  |  |  |
| Group |  |  |  |  |  |  |
| IG, n (%) | 50 (80.6) | 12 (19.4) | 0.33 | 47 (94.0) | 3 (6.0) | NA^a^ |
| CG, n (%) | 54 (87.1) | 8 (12.9) |  | 42 (77.8) | 12 (22.2) |  |
| Age (years), mean (SD) | 39.46 (12.86) | 52.70 (11.18) | **<0.001** | 38.69 (12.24) | 44.07 (15.78) | 0.13 |
| Gender, n (%) |  |  |  |  |  |  |
| Female | 67 (88.2) | 9 (11.8) | 0.10 | 54 (80.6) | 13 (19.4) | NA^a^ |
| Male | 37 (77.1) | 11 (22.9) |  | 35 (94.6) | 2 (5.4) |  |
| Fertility status, N (%) |  |  |  |  |  |  |
| Have child | 62 (76.5) | 19 (23.5) | NA^a^ | 50 (80.6) | 12 (19.4) | NA^a^ |
| No child | 42 (97.7) | 1 (2.3) |  | 39 (92.9) | 3 (7.1) |  |
| Educational background, N (%) |  |  |  |  |  |  |
| Primary and below | 6 (54.5) | 5 (45.5) | **0.018** | 4 (66.7) | 2 (33.3) | NA^a^ |
| Secondary school | 50 (84.7) | 9 (15.3) |  | 43 (86.0) | 7 (14.0) | 0.61^d^ |
| College and above | 48 (88.9) | 6 (11.1) |  | 42 (87.5) | 6 (12.5) |  |
| Living situation, N (%) |  |  |  |  |  |  |
| Living alone | 36 (90.0) | 4 (10.0) | 0.20 | 31 (86.1) | 5 (13.9) | 0.91 |
| Living with others | 68 (81.0) | 16 (19.0) |  | 58 (85.3) | 10 (14.7) |  |
| Occupational status |  |  |  |  |  |  |
| Employed | 66 (89.2) | 8 (10.8) | 0.05 | 56 (84.8) | 10 (15.2) | 0.78 |
| Unemployed | 38 (76.0) | 12 (24.0) |  | 33 (86.8) | 5 (13.2) |  |
| BMI, kg/m^2^, mean (SD) | 22.30 (4.82) | 24.01 (3.39) | 0.13 | 22.12 (4.75) | 23.31 (5.29) | 0.38 |
| BMI category ^b^, N (%) |  |  |  |  |  |  |
| Not overweight or obese | 75 (88.2) | 10 (11.8) | 0.051 | 66 (88.0) | 9 (12.0) | 0.26 |
| Overweight and obese | 29 (74.4) | 10 (25.6) |  | 23 (79.3) | 6 (20.7) |  |
| **Primary outcomes** |  |  |  |  |  |  |
| MVPA (min/week), mean (SD) | 118.02 (83.89) | 156.50 (47.93) | **0.007** | 114.65 (84.67) | 138.00 (78.85) | 0.32 |
| FVC (portion/day), mean (SD) | 4.03 (1.74) | 3.90 (1.47) | 0.76 | 3.92 (1.66) | 4.60 (2.11) | 0.17 |
| Integrated lifestyle indicator **^c^**, N (%) |  |  |  |  |  |  |
| Unhealthy lifestyle | 86 (86.0) | 14 (14.0) | 0.19 | 78 (90.7) | 8 (9.3) | **0.001** |
| Healthy lifestyle | 18 (75.0) | 6 (25.0) |  | 11 (61.1) | 7 (38.9) |  |
| **Secondary outcomes** |  |  |  |  |  |  |
| Internal resources for MVPA, mean (SD) | 2.79 (0.82) | 2.97 (1.01) | 0.38 | 2.76 (0.81) | 2.94 (0.90) | 0.43 |
| External resource for MVPA, mean (SD) | 2.26 (0.98) | 2.75 (1.09) | **0.047** | 2.31 (0.97) | 2.00 (0.98) | 0.26 |
| Internal resources for FVC, mean (SD) | 2.81 (0.82) | 2.84 (1.02) | 0.88 | 2.81 (0.83) | 2.76 (0.78) | 0.81 |
| External resource for FVC, mean (SD) | 2.62 (1.01) | 2.67 (1.16) | 0.85 | 2.67 (1.02) | 2.33 (0.94) | 0.24 |
| Depressive symptoms, mean (SD) | 12.58 (5.04) | 10.70 (4.77) | 0.13 | 12.51 (5.24) | 13.00 (3.90) | 0.73 |
| Perceived quality of life, mean (SD) | 2.81 (0.82) | 2.84 (1.02) | 0.07 | 3.09 (0.85) | 3.18 (0.78) | 0.70 |

Note. IG = intervention group; CG = control group; SD = standard deviation; MVPA = moderate-to-vigorous physical activity; FVC = fruit and vegetable consumption; ^a^ Not applicable as certain cell count <5; ^b^Recategorizing BMI category as “1 = not overweight or obese and 2 = overweight and obese” in order to ensure a meaningful statistical analysis; ^c^Recategorizing integrated lifestyle indicator as “1 = not meet both behavior recommendations and 2 = meet both behavior recommendations (performing ≥ 150 min of MVPA per week and consuming ≥ 5 portions of fruit and vegetables per day)” in order to ensure a meaningful statistical analysis; ^d^ Results of after recategorizing educational background as “1 = secondary school and below, 2 = college and above” in order to ensure a meaningful statistical analysis.
